# Factors Associated with Fluid Sequestration in Patients with Acute Pancreatitis: A Prospective Study in Tertiary Centre Hospital in Nepal

**DOI:** 10.1155/2021/5579267

**Published:** 2021-06-24

**Authors:** Raju Bhandari, Krishna Sapkota, Seema Subedi, Som Kumar Shrestha, Edward Sutanto, Prabhat Jha, Ramesh Singh Bhandari

**Affiliations:** ^1^Department of General and Gastrointestinal Surgery, Tribhuvan University Teaching Hospital, Institute of Medicine, Tribhuvan University, Kathmandu, Nepal; ^2^Public Health Officer, Health Coordination Division, Ministry of Health and Population, Government of Nepal, Nepal; ^3^Global Disease Epidemiology and Control, Department of International Health, Johns Hopkins Bloomberg School of Public Health, Baltimore, USA; ^4^Department of Medical Statistics, London School of Hygiene and Tropical Medicine, London, UK; ^5^Department of Health Behavior, Roswell Park Comprehensive Cancer Center, Buffalo, USA

## Abstract

**Background:**

Acute pancreatitis (AP) is associated with extensive fluid sequestration. The aim of this study was to determine association of fluid sequestration at 48 hours after hospital admission (FS^48^) in AP patients with demographics, clinical parameters, and outcomes of AP.

**Methods:**

A prospective observational study was carried out on all adult patients with AP admitted to Tribhuvan University Teaching Hospital, Nepal, from January to September 2017. FS^48^ was calculated as the difference between fluid input and output in the first 48 hours of admission. The Kruskal-Wallis test with post hoc Dunn's test examined the difference in FS^48^ between mild AP, moderately severe AP, and severe AP. Linear regression analysis was used to evaluate association between FS^48^ with patients' characteristics and outcomes of AP. Outcomes of AP assessed included pancreatic necrosis, persistent organ failure, length of stay, and in-hospital mortality.

**Results:**

Eighty patients (median age 44 years; 57% male) with a median FS^48^ of 1610 mL were evaluated. The median FS^48^ for mild AP, moderately severe AP, and severe AP were 1,180 mL, 2,380 mL, and 3,500 mL, respectively. There was a significant difference in pairwise comparisons between mild AP and moderately severe AP, along with mild AP and severe AP. Younger age, other etiology, and higher creatinine were independently associated with increased FS^48^. Increased FS^48^ was significantly associated with pancreatic necrosis, persistent organ failure, and in-hospital mortality.

**Conclusions:**

In our study population, younger age and higher creatinine were predictors of increased FS^48^. Increased FS^48^ was associated with poorer outcomes of AP.

## 1. Introduction

Acute pancreatitis (AP), a common surgical presentation, is the most common pancreatic disease with global estimates of incidence and mortality being 33.7 cases per 100,000 person-years and 1.6 deaths per 100,000 person years, respectively [1]. Gallstones and alcohol are the two most common etiological factors for AP, accounting approximately 80% of all causes, with gallstone pancreatitis being twice as frequent as alcohol pancreatitis [2–4].

Based on the revised Atlanta classification, AP is categorized into mild, moderately severe, and severe [5]. Overall, mortality increased with disease severity, ranging from less than 5% in mild AP up to 17.8%–41.9% in severe AP [6, 7]. Supportive care, which includes fluid therapy, bowel rest, and analgesics, is the mainstay of treatment in AP [8, 9]. As fluid sequestration (FS) is commonly seen in patients with AP and associated with worse outcome [10, 11], identification of predictors of FS may help clinician to optimize fluid resuscitation among patients with AP that requires early and aggressive fluid therapy.

Two studies (de-Madaria et al. and Sinha et al.) have examined early predictors of FS in AP [10, 11]. In both studies, younger age, alcoholic etiology, hemoconcentration, and systemic inflammatory response syndrome (SIRS) were found to be independent predictors of increased fluid sequestration at 48 hours after hospital admission (FS^48^) [10, 11]. Additionally, hyperglycemia was found to be a significant predictor of FS^48^ in one study [10]. These retrospective studies however were conducted in developed countries [10, 11], yet AP in the developing countries may have a markedly different disease profile to that in developed countries [12, 13]. While alcohol consumption is a leading cause of AP in developed countries, other causes of AP (such as hypertriglyceridemia or mumps) are more prominent in developing countries [12, 13], thus requiring different management to treat underlying causes of AP. Thus, we primarily aimed to validate the association between characteristics of AP patients at admission and FS^48^ in a developing country setting. Secondarily, we also examined the association between FS^48^ and outcomes of AP among these patients, along with the difference of FS^48^ between mild, moderately severe, and severe AP. We used the STROBE statement to guide the writing of this study [14].

## 2. Materials and Methods

### 2.1. Patient Population

We conducted a prospective cohort study of adult patients (age ≥ 18 years) with a diagnosis of AP admitted in the surgical wards of Tribhuvan University Teaching Hospital, Nepal, from January to September 2017. In accordance with the revised Atlanta classification, AP was defined as 2 of the following 3 criteria: (1) characteristic abdominal pain, (2) serum amylase and/or lipase greater than 3 times the upper limit of normal, and (3) abdominal imaging findings consistent with AP [5]. We excluded patients undergoing hemodialysis, patients with congestive heart failure, and transferred patients with incomplete record on FS^48^.

### 2.2. Variables

The main dependent variable in this study is FS^48^. Similar with previous studies [10, 11], we calculated FS^48^ as the difference between the total fluid input and total fluid output in the first 48 hours of admission. Fluid input included administration of all intravenous crystalloid or colloid preparations, blood products, antibiotics, and oral fluid intake. Fluid output included recorded volumes of vomitus, urine, stool, and insensible losses (10 mg per kg body weight per day). Additionally, 500 mL was added to the fluid output if patients had a temperature > 37.8°C per day.

Demographic characteristics (age and sex) and clinical parameters (etiology, SIRS at presentation, hematocrit, total leukocyte count, serum glucose, serum creatinine, blood urea nitrogen (BUN), and serum sodium) were collected. Etiology was categorized into alcohol, gallstones, and other. As it is possible for patient to have more than one etiology of AP, etiology was treated as a nonmutually exclusive categorical variable. SIRS is defined as the occurrence by two or more of the following conditions: (1) temperature > 38°C or <36°C, (2) heart rate > 90 beats per minute, (3) respiratory rate > 20 breaths per minute, and (4) white blood cell count > 12,000/mm^3^, <4,000/mm^3^, or >10% immature (band) forms [15].

We also collected data on complications of AP (persistent organ failure and pancreatic necrosis), in-hospital mortality, and length of stay. Persistent organ failure (POF) was defined as a Marshall score of 2 or greater in the renal, pulmonary, and or cardiovascular system for longer than 48 hours [5]. Pancreatic necrosis was defined according to the revised Atlanta classification [5] and evaluated using abdominal contrast-enhanced computed tomography (CECT). Abdominal CECT was indicated in the following situations in this study: (1) if there is diagnostic uncertainty; (2) to distinguish interstitial from necrotizing pancreatitis in patients with severe AP; (3) in patients with organ failure, a sign of sepsis or progressive clinical deterioration; and (4) when a localized complication (such as fluid collection, pseudocyst, or pseudoaneurysm) is suspected [16]. We classified patients based on severity of AP into mild AP (MAP), moderately severe AP (MSAP), and severe AP (SAP) in accordance to the revised Atlanta classification [5].

### 2.3. Study Endpoint

The primary study endpoint is FS^48^ outcome, while the secondary study endpoints are POF, pancreatic necrosis, in-hospital mortality, and length of stay outcomes.

### 2.4. Statistical Analysis

Demographic characteristics, clinical parameters, and outcomes of AP were reported using descriptive statistics. Three separate analyses were done in accordance to our aims. First, as FS^48^ did not follow normal distribution, the Kruskal-Wallis test was employed to examine differences in FS^48^ between MAP, MSAP, and SAP. Multiple pairwise comparisons were addressed by using Dunn's test. Second, simple and multiple linear regressions were used to evaluate the association between potential predictors at admission and FS^48^. Third, simple and multiple linear regressions were used to evaluate the association between FS^48^ and outcomes of AP. In both regression analyses, we reported *β* coefficient, which represented change (mL) in FS^48^ associated with unit change in predictor variables, and 95% confidence interval. All statistical analyses were performed in Stata version 15.0 (StataCorp, College Station, Texas, United States). All tests were two-tailed and considered significant at *p* < 0.05.

## 3. Results

A total of 80 patients were recruited in this study.  Table 1 describes baseline characteristics and outcomes in our study cohort. The median age was 44 years, and majority were male (57.5%). Gallstone was the most common etiology (80.0%). Alcohol etiology was present in approximately a quarter of study cohort (22.5%), while other etiology (3 cases) included one each of idiopathic, pancreatic malignancy, and hypertriglyceridemia. Nearly one-fifth developed persistent organ failure and 2.5% died during hospitalization. Abdominal contrast-enhanced computed tomography was performed in 23 patients to evaluate pancreatic necrosis. Among these 23 patients, almost half have a degree of pancreatic necrosis (47.8%). The median value of FS^48^ was 1610 mL (interquartile range (IQR): 810-3575 mL).

**Figure 1:**
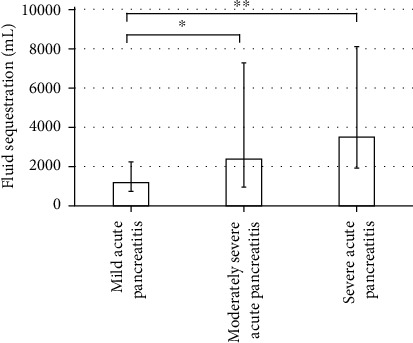
Fluid sequestration in the first 48 hours from admission between mild, moderately severe, and severe acute pancreatitis. The values represent median and error bars are interquartile range. ^∗^*p* < 0.05 and ^∗∗^*p* < 0.01 (Dunn's test).

Figure 1 shows the difference in FS^48^ between MAP, MSAP, and SAP. The median FS^48^ for MAP, MSAP, and SAP were 1180 mL (IQR: 730-2240 mL), 2380 mL (IQR: 950-7280 mL), and 3500 mL (IQR: 1920-8110 mL), respectively. The Kruskal-Wallis test showed there was a significant difference in FS^48^ between these three groups (*p* < 0.01). Post hoc analysis showed that there was significant difference for pairwise comparisons of MAP versus MSAP (*p* < 0.05) and MAP versus SAP (*p* < 0.01).

Table 2 displays the association between patient characteristics and FS^48^. Younger age, other etiology, and higher creatinine were independently associated with increased FS^48^.  Table 3 displays the association between FS^48^ and outcomes of AP. Although length of stay was not associated with FS^48^, FS^48^ was associated with pancreatic necrosis, persistent organ failure, and in-hospital mortality.

## 4. Discussions

This prospective study evaluated predictors and outcomes associated with increased FS^48^ among 80 patients with AP in a developing country setting. There are three key findings in this study. First, there is a significant increase in median FS^48^ in MSAP and SAP compared to MAP. Second, we found that younger age, other etiology, and higher creatinine predict increased FS^48^. Third, increased FS^48^ is associated with worse patient outcomes.

Previous study by Ranson et al. in 1974 reported a mean FS^48^ of 3.7 L and 5.6 L for MAP and SAP, respectively [17]. At the time of publication of the study, there was no classification of MSAP for severity of AP. A study by de-Madaria et al. reported the median FS^48^ was 3.0 L (IQR: 1.5–5.0 L) and 6.4 L (IQR: 3.6-9.5 L) in those without necrosis and those with necrosis [10], while a study by Sinha et al. reported median FS was 4.0 L (IQR: 2.0-5.9 L) and 9.2 L (IQR: 4.8-13.2 L) in those without POF and those with POF [11]. While both studies did not report median FS^48^ based on severity of AP specifically, both necrosis and POF are criteria for MSAP and SAP [5]. Thus, our finding that there is a significant increase in median FS^48^ in MSAP and SAP compared to MAP is consistent with previous studies.

Although studies on AP have been conducted in Nepal, there is no study that has examined FS among patients with AP. A previous study from our center noted biochemical markers, total serum calcium and albumin-corrected calcium, are useful severity predictors in AP [18]. Our study further adds that median FS^48^ is a useful parameter as increased median FS^48^ was observed in more severe AP. SAP has been noted to be associated with vascular leak syndrome [19], which increased systemic vascular permeability leading to extravasation of fluids and proteins into tissues, thus increasing FS.

Consistent with previous studies [10, 11], our study confirms that younger age is associated with increased FS^48^. It is hypothesized that, due to concern of volume overload, older patients may receive less aggressive fluid resuscitation resulting in lower FS [10]. While previous studies found alcohol etiology to be predictors of increased FS^48^ [10, 11], we found other etiologies of AP to be significantly associated with increased FS^48^. Additionally, we did not find hematocrit, glucose, and presence of SIRS to be predictors of increased FS^48^ in our study cohort. These discrepancies may be explained partly due to differences between our study cohort and patients from previous studies. Unlike our study where majority of AP etiology was gallstones, both previous studies have a markedly lower gallstones etiology (de-Madaria et al.: 41.4% and Sinha et al.: 13.2%) [10, 11]. Secondly, our study was conducted in low-resource setting which influenced on medical imaging modalities performed to our study cohort. Lastly, our relatively small sample size may not allow us to have enough power to detect difference in increased FS^48^.

Higher creatinine was found to be independent predictors of increased FS^48^ in our study cohort. Both creatinine and BUN are well-known markers of renal function. Creatinine, however, have been hypothesized to be less sensitive to small changes in intravascular volume and better reflect visceral organ injury [20]. As kidney injury is a common complication of AP, with prevalence around 20% in all AP patients and up to 70% in SAP, it can be resulted from volume depletion due to fluid sequestration [21, 22]. Multiple studies also have demonstrated that elevated serum creatinine level is associated with the development of pancreatic necrosis, POF, and mortality [20, 23, 24].

We found FS^48^ to be significantly associated with several outcomes of AP, including POF, pancreatic necrosis, and in-hospital mortality, but not with increased length of stay. While FS^48^ was significantly associated with POF consistently across studies [10, 11], there is a mixed result for pancreatic necrosis and length of stay. It is important to note that only the presence of POF is used to define severe AP, and studies have reported that POF was the strongest predictor of mortality in necrotizing pancreatitis [5, 25]. While de-Madaria's study reported increased FS^48^ was associated with pancreatic necrosis and length of stay, Sinha et al. did not find these associations [10, 11]. This mixed result may be attributed to the difference rates of these complications occurring in each study cohort. Our study also reinforces finding from a previous study that fluid sequestration of 2 L or more per day, and lasting longer than 48 hour, is an accurate and simple predictor of mortality in AP [26].

To the best of our knowledge, this is the first study from a developing country that examined association between increased FS^48^, patients' characteristics, and outcomes of AP. While the prospective nature of this study is a strength, there are several limitations in this study. First, the sample size is relatively small. Although we included all eligible patients for 9 months, we were only able to recruit 80 patients to our study cohort. Second, the study was conducted in a single tertiary hospital in Nepal. As our center is a major referral hospital in Nepal, more severe AP patients were recruited in this study; thus, it may limit the generalizability of our findings. Lastly, similar with Sinha et al.'s study [11], we also did not perform abdominal CECT in all patients; thus, the rates of pancreatic necrosis or acute fluid collection may be underreported. However, abdominal CECT is not routinely indicated in AP management as unnecessary imaging studies, especially in the early hospital course, is associated with increased radiation dose for patients and high health care costs, frequently without impact on patient outcomes or management [16, 27–29]. Nevertheless, our study provides an important external validation of the results from de-Madaria et al.'s and Sinha et al.'s studies in the context of low-resource settings.

## 5. Conclusions

In conclusion, younger age and higher creatinine were predictors of increased FS^48^ in our study cohort. As increased FS^48^ was associated with poorer outcomes of AP, there is a need to develop a simple scoring system that incorporates easily obtained variables at presentation to reliably predict FS for AP patients.

## Figures and Tables

**Table 1:** Demographics characteristics, clinical parameters, and outcomes of the study cohort.

	*N* = 80
Demographics	
Age (years)	44 (36-56.5)
Male	46 (57.5%)
Clinical characteristics	
FS^48^ (mL)	1610 (810-3575)
Etiology^∗^	
Alcohol	18 (22.5%)
Gallstones	64 (80.0%)
Others	3 (3.7%)
SIRS at presentation	34 (42.5%)
Hematocrit (%)	40.0 (35.2-44.5)
Total leukocyte count (/mm^3^)	11350 (8000-14750)
Glucose (mg/dL)	143.1 (111.6-173.7)
Creatinine (mg/dL)	0.91 (0.78-1.33)
BUN (mmol/L)	5.0 (3.5-9.1)
Sodium (mmol/L)	138.3 (135.0-141.5)
Outcomes	
Pancreatic necrosis (*n* = 23)	
None	12 (52.2%)
≤30	7 (30.4%)
>30	4 (17.4%)
Persistent organ failure	15 (18.8%)
In-hospital mortality	2 (2.5%)
Length of stay (days)	4.5 (3.0-8.5)
Severity of acute pancreatitis	
Mild	54 (67.5%)
Moderately severe	11 (13.7%)
Severe	15 (18.8%)

Values are expressed in median (interquartile range) or *n* (%). ^∗^Add up to more than 100% as more than one etiology may be identified in a patient. Abbreviations: BUN: blood urea nitrogen; FS^48^: fluid sequestration at 48 hours after hospital admission; SIRS: systemic inflammatory response syndrome.

**Table 2:** Association between demographic characteristics and clinical parameters with fluid sequestration in the first 48 hours from admission.

Variables	Unadjusted *β* coefficient (95% CI)	Adjusted *β* coefficient (95% CI)
Demographics		
Age (years)	-20.8 (-58.8, 17.1)	-45.2 (-72.1, -18.4)^∗∗^
Female	-196.6 (-1392.3, 999.0)	-1.5 (-952.9, 949.9)
Clinical characteristics		
Alcohol etiology	-1520.6 (-2894.9, -146.3)^∗^	-754.5 (-1954.4, 445.4)
Gallstones etiology	1379.1 (-66.6, -2824.7)	589.5 (-609.1, 1788.1)
Other etiology	3411.1 (394.3, 6427.9)^∗^	4591.4 (2474.0, 6708.8)^∗∗∗^
SIRS at presentation	1037.1 (-136.3, 2210.5)	21.4 (-824.9, 867.7)
Hematocrit (%)	100.9 (8.3, 193.5)^∗^	51.9 (-17.8, 121.7)
Glucose (mg/dL)	10.0 (0.5, 19.5)^∗^	2.9 (-4.4, 10.2)
Creatinine (mg/dL)	3124.6 (2333.2, 3916.1)^∗∗∗^	2943.8 (2061.7, 3825.9)^∗∗∗^
BUN (mmol/L)	174.0 (58.8, 289.3)^∗∗^	-3.7 (-101.2, 93.7)
Sodium (mmol/L)	97.8 (-20.4, 215.9)	43.5 (-38.6, 125.7)

Abbreviations: BUN: blood urea nitrogen; CI: confidence interval; SIRS: systemic inflammatory response syndrome. ^∗^*p* < 0.05, ^∗∗^*p* < 0.01, and ^∗∗∗^*p* < 0.001.

**Table 3:** Association between outcomes of AP and FS in the first 48 hours from admission.

Variables	Unadjusted *β* coefficient (95% CI)	Adjusted *β* coefficient (95% CI)
Pancreatic necrosis		
None	Ref	Ref
≤30	2270.4 (261.7, 4279.0)^∗^	4182.0 (1373.0, 6990.9)^∗∗^
>30	4035.0 (1596.6, 6473.4)^∗∗^	6088.7 (3683.4, 8494.1)^∗∗∗^
Persistent organ failure		
No	Ref	Ref
Yes	1893.6 (439.6, 3347.6)^∗^	-3192.1 (-5571.2, -813.0)^∗^
In-hospital mortality		
No	Ref	Ref
Yes	6076.7 (2544.7, 9608.9)^∗∗^	5956.5 (2523.1, 9389.9)^∗∗^
Length of stay	101.4 (19.7, 183.0)^∗^	19.8 (-64.8, 104.8)

^∗^ *p* < 0.05, ^∗∗^*p* < 0.01, and ^∗∗∗^*p* < 0.001.

## Data Availability

The dataset used for the current study is available from the corresponding author on reasonable request.

## Abbreviations

AP: Acute pancreatitis
BUN: Blood urea nitrogen
CECT: Contrast-enhanced computed tomography
CI: Confidence interval
FS^48^: Fluid sequestration in the first 48 hours from admission
IQR: Interquartile range
MAP: Mild acute pancreatitis
MSAP: Moderately severe acute pancreatitis
SAP: Severe acute pancreatitis
SIRS: Systemic inflammatory response syndrome.

## References

[1] Xiao A. Y., Tan M. L. Y., Wu L. M. (2016). Global incidence and mortality of pancreatic diseases: a systematic review, meta-analysis, and meta-regression of population-based cohort studies. *The Lancet Gastroenterology & Hepatology*.

[2] Zilio M. B., Eyff T. F., Azeredo-Da-Silva A. L. F., Bersch V. P., Osvaldt A. B. (2019). A systematic review and meta-analysis of the aetiology of acute pancreatitis.

[3] Sakorafas G. H., Tsiotou A. G. (2000). Etiology and pathogenesis of acute pancreatitis: current concepts. *Journal of Clinical Gastroenterology*.

[4] Nesvaderani M., Eslick G. D., Vagg D., Faraj S., Cox M. R. (2015). Epidemiology, aetiology and outcomes of acute pancreatitis: a retrospective cohort study. *International Journal of Surgery*.

[5] Banks P. A., Bollen T. L., Dervenis C. (2013). Classification of acute pancreatitis -2012: revision of the Atlanta classification and definitions by international consensus. *Gut*.

[6] Sarri G., Guo Y., Iheanacho I., Puelles J. (2019). Moderately severe and severe acute pancreatitis: a systematic review of the outcomes in the USA and European Union-5. *BMJ Open Gastroenterology*.

[7] Gravante G., Garcea G., Ong S. L. (2009). Prediction of mortality in acute pancreatitis: a systematic review of the published evidence. *Pancreatology*.

[8] Tenner S., Baillie J., Dewitt J., Vege S. S. (2013). American College of Gastroenterology guideline: management of acute pancreatitis. *The American Journal of Gastroenterology*.

[9] Greenberg J. A., Hsu J., Bawazeer M. (2016). Clinical practice guideline: management of acute pancreatitis. *Canadian Journal of Surgery*.

[10] de-Madaria E., Banks P. A., Moya-Hoyo N. (2014). Early factors associated with fluid sequestration and outcomes of patients with acute pancreatitis. *Clinical Gastroenterology and Hepatology*.

[11] Sinha A., Vázquez N. Q., Faghih M. (2016). Early predictors of fluid sequestration in acute pancreatitis: a validation study. *Pancreas*.

[12] Ahmed K. U., Ahad M. A., Alim M. A., Ekram A. S. (2017). Clinical profile of acute pancreatitis in a teaching hospital. *Bangladesh Medical Journal Khulna*.

[13] Fan J., Ding L., Lu Y., Zheng J., Zeng Y., Huang C. (2018). Epidemiology and etiology of acute pancreatitis in urban and suburban areas in Shanghai: a retrospective study. *Gastroenterology Research and Practice*.

[14] Vandenbroucke J. P., von Elm E., Altman D. G. (2014). Strengthening the Reporting of Observational Studies in Epidemiology (STROBE): explanation and elaboration. *International Journal of Surgery*.

[15] Bone R. C., Balk R. A., Cerra F. B. (1992). Definitions for sepsis and organ failure and guidelines for the use of innovative therapies in sepsis. *Chest*.

[16] Williams N. S., Bulstrod C. J. K., O'Connell P. R. (2014). *Acute and chronic pancreatitis. Bailey & Love's Short Practice of Surgery. 26th edition*.

[17] Ranson J. H., Rifkind K. M., Roses D. F., Fink S. D., Eng K., Spencer F. C. (1974). Prognostic signs and the role of operative management in acute pancreatitis. *Surgery, Gynecology & Obstetrics*.

[18] Pokharel A., Sigdel P. R., Phuyal S., Kansakar P. B. S., Vaidya P. (2017). Prediction of severity of acute pancreatitis using total serum calcium and albumin-corrected calcium: a prospective study in tertiary center hospital in Nepal. *Surgery Research and Practice*.

[19] Whitcomb D. C., Muddana V., Langmead C. J. (2010). Angiopoietin-2, a regulator of vascular permeability in inflammation, is associated with persistent organ failure in patients with acute pancreatitis from the United States and Germany. *The American Journal of Gastroenterology*.

[20] Muddana V., Whitcomb D. C., Khalid A., Slivka A., Papachristou G. I. (2009). Elevated serum creatinine as a marker of pancreatic necrosis in acute pancreatitis. *The American Journal of Gastroenterology*.

[21] Wajda J., Dumnicka P., Maraj M., Ceranowicz P., Kuźniewski M., Kuśnierz-Cabala B. (2019). Potential prognostic markers of acute kidney injury in the early phase of acute pancreatitis. *International Journal of Molecular Sciences*.

[22] Lattanzio M. R., Kopyt N. P. (2009). Acute kidney injury: new concepts in definition, diagnosis, pathophysiology, and treatment. *The Journal of the American Osteopathic Association*.

[23] Lipinski M., Rydzewski A., Rydzewska G. (2013). Early changes in serum creatinine level and estimated glomerular filtration rate predict pancreatic necrosis and mortality in acute pancreatitis: creatinine and eGFR in acute pancreatitis. *Pancreatology*.

[24] Wan J., Shu W., He W. (2019). Serum creatinine level and APACHE-II score within 24 h of admission are effective for predicting persistent organ failure in acute pancreatitis. *Gastroenterology Research and Practice*.

[25] Guo Q., Li A., Xia Q. (2014). The role of organ failure and infection in necrotizing pancreatitis: a prospective study. *Annals of Surgery*.

[26] Sauven P., Playforth M. J., Evans M., Pollock A. V. (1986). Fluid sequestration: an early indicator of mortality in acute pancreatitis. *The British Journal of Surgery*.

[27] Mortele K. J., Ip I. K., Wu B. U., Conwell D. L., Banks P. A., Khorasani R. (2011). Acute pancreatitis: imaging utilization practices in an urban teaching hospital—analysis of trends with assessment of independent predictors in correlation with patient outcomes. *Radiology*.

[28] Spanier B. W., Nio Y., van der Hulst R. W., Tuynman H. A., Dijkgraaf M. G., Bruno M. J. (2010). Practice and yield of early CT scan in acute pancreatitis: a Dutch observational multicenter study. *Pancreatology*.

[29] Rocha A. P. C., Schawkat K., Mortele K. J. (2020). Imaging guidelines for acute pancreatitis: when and when not to image. *Abdominal Radiology*.

